# Supplementary material for: Confounding adjustment performance of ordinal analysis methods in stroke studies
Source: PLoS One. 2020 Apr 16;15(4):e0231670. doi: 10.1371/journal.pone.0231670 (PMC7162480; doi:10.1371/journal.pone.0231670)
Supplement: S1 Appendix — (DOCX) [file pone.0231670.s001.docx]

**Appendix 1**. Data generation process.

We generated a seven-step ordinal outcome variable (*y*), on which a dichotomous exposure variable (*x_e_*) had no effect, whereas confounding variables (x*_1_,…,x_5_*) did. We did not model a relationship or correlation between the confounding variables. In order to be able to create *y*, we first created a latent variable (*y**), which reflects the presence of confounding:(12)

*y*_­_ =* $\sum_{j=1}^{5} \beta_{j}x_{ji}+\varepsilon_{i}$*, with ε_i_ ~N(0,1)*

In which *x_1i_,…,x_5i_* are the confounding variables for patient i and *β_1_,…, β_5­_* are the regression coefficients of these variables. The term ε_i_ denotes the error term, which varies per subject. Thus, observations with more confounding present (i.e. more exposed to dichotomous confounding present or with higher values for continuous confounding variables) generally have higher overall risk values.

Out of *y**, we created six variables (*y*_1_* - *y*_6_*), that represented the six cut-off points between the seven outcome categories:

*y*_1-6­_ = std(y*) + invnormal(α_1-6_)*

We standardized *y** so that we could shift the placement of the latent variable distributions accurately, by adding the inverse normal (invnormal) of values between 0 and 1 (*α­­­_1-6_*). For *α_1-6_* we used 1/7, … , 6/7, as we aimed to generate a uniform distribution of *y*. In other words, *α_1-6_* are the fractions of observations we aim to keep below each cut-off point of the mRS. Thus, every observation had six latent variables (*y*_1_* - *y*_6_*), which only differed in the addition of the normal sextiles. Finally, we generated the ordinal outcome *y* based on the six latent variables created from *y**

*if y*_1­_ > 0 then y = 6, if y*_2_ > 0 then y = 5, …, if y*_6_ > 0 then y = 1, else = 0*

This states, that if condition *y*_1­_ > 0* for an observation holds true, then *y* = 6. If *y*_1­_ > 0* is false, it checks whether *y*_2_ > 0* holds true. *If y*_2­_ > 0* is true, then *y* = 5, et cetera. If the last condition (*y*_6_ > 0*) is false, then *y* = 0. So, observations with *y* = 6, have a positive *y*_1_*_­_ despite adding a negative term to the equation. At the other end of the spectrum, observations with y = 0 have a *y*_6_* smaller than 0, despite adding a positive term. So, in short, observations with more confounding present are more likely to have higher *y**, and are therefore more likely to end up in higher categories of *y*.

To complete the requirement for confounding, the exposure also needed to be dependent on the confounding variables. Therefore, we constructed the binary exposure (X_e_) based on whether the confounding variable terms (*β­_c1-ci_ x_c1-ci_ - β­_c1-ci_ x_c1-ci(mean)_*) and a base risk of 0.5 are higher than a randomly generated number between 0 and 1. Here, we subtracted the mean of each confounding variable by its regression coefficient to balance the equation around 0.5, and thus a similar number of cases exposed in each run and scenario.
